# Cerebral Salt-Wasting Syndrome in a Patient With Active Pulmonary Tuberculosis

**DOI:** 10.7759/cureus.21202

**Published:** 2022-01-13

**Authors:** Waqas Memon, Ayesha Akram, Karishma Popli, James B Spriggs, Sana Rehman, Graham Gipson, Todd Gehr

**Affiliations:** 1 Internal Medicine/Nephrology, University of Virginia, Lynchburg, USA; 2 Internal Medicine, Combined Military Hospital, Rawalpindi, PAK; 3 Internal Medicine, Rawalpindi Medical University, Rawalpindi, PAK; 4 Internal Medicine, Virginia Commonwealth University School of Medicine, Richmond, USA; 5 Psychiatry and Behavioral Sciences, Johns Hopkins Bayview Medical Center, Bayview, USA; 6 Medicine, Islamic International Medical College, Islamabad, PAK; 7 Nephrology, Virginia Commonwealth University, Richmond, USA

**Keywords:** cerebral salt-wasting syndrome, active pulmonary tuberculosis, diabetes insipidus, siadh, nephrology

## Abstract

A 37-year-old female with a medical history of recently diagnosed active pulmonary tuberculosis and a new intracranial lesion presented with altered mental status, nausea, and vomiting for two days. An initial physical examination revealed that the patient was euvolemic. Laboratory findings revealed a serum sodium concentration of 105 mEq/L. During her admission, she was initially managed with lactated ringer solution in the emergency department, followed by 3% normal saline in the intensive care unit, and, eventually, on oral sodium chloride and fluid restriction on discharge. Once she was stabilized, she had episodes of dizziness, and concerns were raised about the salt-wasting syndrome.

## Introduction

Cerebral salt wasting (CSW) is a potential cause of hyponatremia in patients with neurologic disease, most commonly in those with subarachnoid hemorrhage. CSW was first described in 1950 by Peters et al. who reported three patients with intracranial disorders and associated hypovolemic hyponatremia and clinical symptoms of diuresis, natriuresis, and dehydration [1]. Both CSW and syndrome of inappropriate antidiuretic hormone (SIADH) have similar features and laboratory signs but different treatment regimens, making diagnosis and successful management critical. CSW is diagnosed in patients who have evidence of hypovolemia, hyponatremia with low plasma osmolality, and inappropriately elevated urine osmolality, as well as an elevated urine sodium concentration with evidence of net negative sodium balance and low serum uric acid concentration due to urate wasting in urine [2].

Although the etiology of CSW is not well understood, it can develop as a protective mechanism in response to an excessive rise in intracranial pressure, vasospasm in subarachnoid hemorrhage, or meningitis [3]. One hypothesis suggests that natriuretic factors such as brain natriuretic peptide (BNP) are involved, causing impairment in renal tubular sodium reabsorption and inhibition of renin release leading to an increase in sodium excretion and urine volume [4].

Here, we present the case of a 37-year-old female who presented with altered mental status (AMS), nausea, and vomiting for two days. On presentation, she was found to have a serum sodium concentration of 105 mEq/L.

## Case presentation

**Figure 1:**
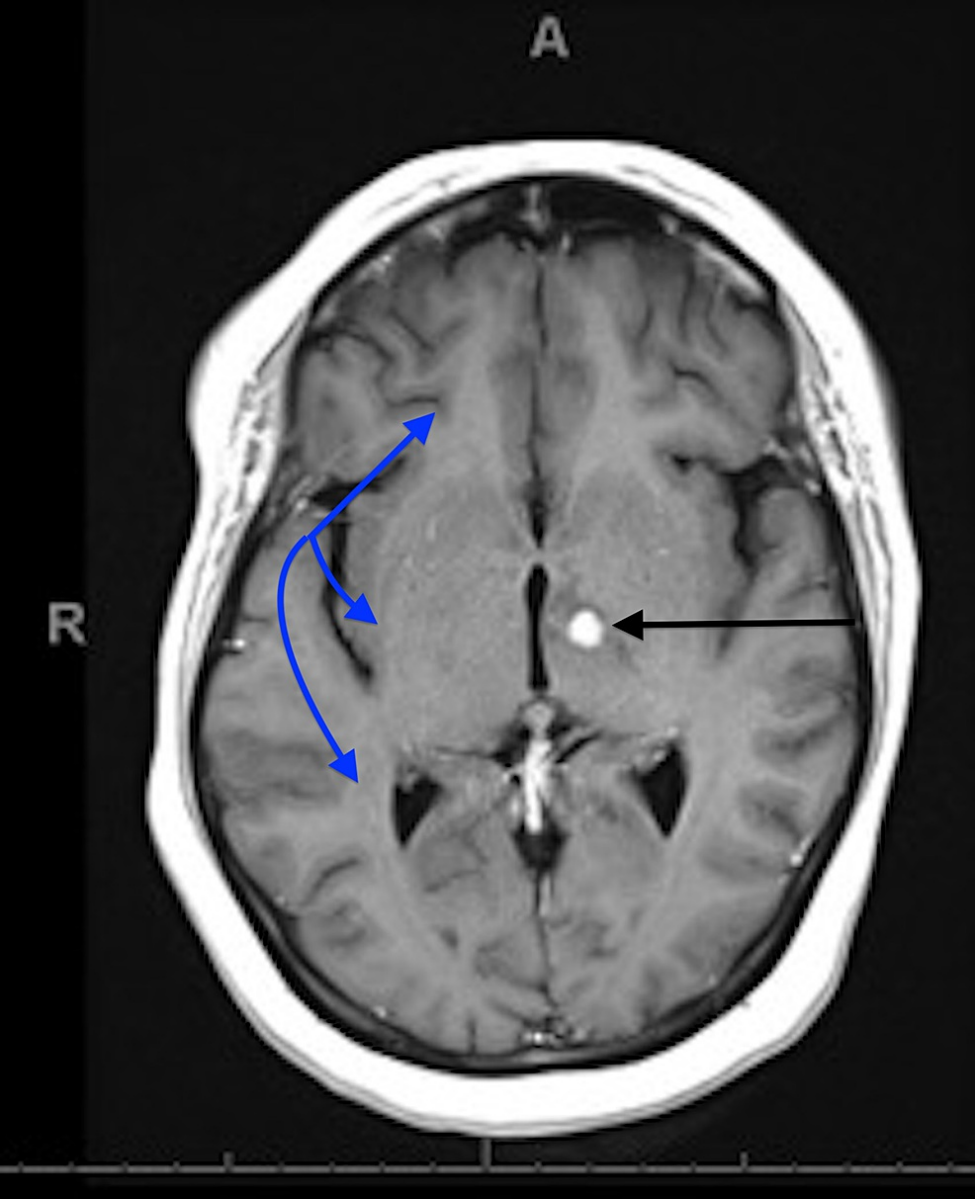
MRI of the brain with and without contrast. Black arrow: A well-circumscribed homogeneously enhancing nodule in the left midbrain/thalamic junction measuring approximately 7 × 7 mm, which previously measured 9 × 9 mm. Blue arrow: A small amount of surrounding vasogenic edema extending into the posterior limb of the internal capsule, left thalamus, and left midbrain. There is no significant midline shift and no transtentorial herniation. There is no associated hemorrhage or diffusion restriction. MRI: magnetic resonance imaging

A 37-year-old female with a medical history of recently diagnosed active pulmonary tuberculosis (TB), right lower lung mass (status post wedge resection), and a new intracranial lesion suspected to be due to TB presented with AMS, nausea, and vomiting for two days. On presentation, her blood pressure was 145/81 mmHg, pulse was 81 beats per minute, and saturation was 93-94% on room air. She was found to have a serum sodium concentration of 105 mEq/L. In the emergency department, she received 1 L of lactated Ringer’s solution, after which her serum sodium concentration decreased to 101 mEq/L. She underwent an MRI (Figure 1) due to her confused mental status. The MRI revealed no significant midline shift, transtentorial herniation, associated hemorrhage, or diffusion restriction. Other causes of hyponatremia, including adrenocorticotropic hormone (ACTH) and thyroid-stimulating hormone (TSH), were within normal limits.

Upon admission, serum osmolality was 214 mOsm/kg, urine osmolality was 657 mOsm/kg, urine potassium concentration was 121.9 mEq/L, and urine sodium concentration was 163 mmol/L. She received a 100 mL infusion of 3% saline, following which her serum sodium concentration increased to 109 mEq/L. In an attempt to stop overcorrection, DDAVP 2 µg was given; serum sodium concentration on the next check was 102 mEq/L. She received another 100 mL infusion of 3% normal saline, and repeat urine chemistries were as follows: urine sodium concentration 48 mEq/L (normal value: 40-220 mEq/L); urine potassium concentration 26 mEq/L (normal value 25-125 mEq/L); urine osmolality 434 mOsm/kg (normal value: 500-850 mOsm/kg). Ultimately, urine sodium concentration increased to 112 meq/L, and 3% saline was discontinued. Her serum sodium again increased to 119 mEq/L, and she was started on 5% dextrose in water at 50 mL/hour with a goal serum sodium concentration of 120 mEq/L. After achieving a serum sodium level of 121 mEq/L, the patient was started on oral urea (ure-Na™), 15 g twice daily, with instructions to increase dietary protein intake and limit oral fluid intake to less than 1 L/day.

**Figure 2:**
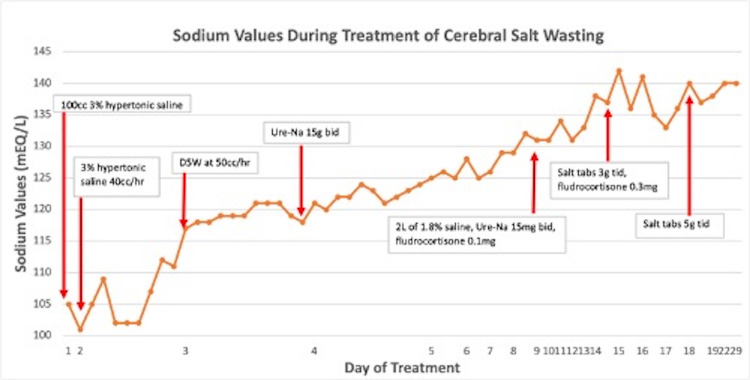
Sodium values during the treatment of cerebral salt wasting.

Once transferred out of the intensive care unit, she was initially continued on oral fluid restriction and oral urea. However, she later had episodes of dizziness, and concerns were raised about the salt-wasting syndrome. Her 24-hour urine sodium excretion rate was very high at 250. She was started on oral sodium chloride, 5 g thrice daily. Based on an analysis of her total body sodium balance over several days, she received additional sodium expansion with 1.8% saline. She was also started on fludrocortisone, 0.1 mg once daily. She was discharged in stable condition on oral fludrocortisone, 0.3 mg once daily, and oral sodium chloride, 5 g thrice daily. Serum sodium with hospital course and treatment is presented in Figure 2 and Table 1.

**Table 1:** Comparison between the day of admission and sodium value and intervention. On days two, three, and four, serum sodium levels were measured every two to three hours. On day five, serum sodium level was measured every eight hours. On subsequent days, the levels were measured either once daily or every 12 hours.

Day	Sodium value (mEq/L)	Intervention done
1	105	100 cc 3% hypertonic saline
2	101	3% hypertonic saline 40 cc/hour, goal sodium concentration of 112 mEq/L
2	105	
2	109	
2	102	
2	102	
2	102	
2	107	
2	112	
2	111	
3	117	D5W at 50 cc/hour, goal sodium concentration of 120 mEq/L
3	118	
3	118	
3	119	
3	119	
3	119	
3	121	
3	121	
3	121	
3	119	
3	118	
4	121	Ure-Na 15 g bid
4	120	
4	122	
4	122	
4	124	
4	123	
4	121	
4	122	
4	123	
4	124	
5	125	
5	126	
5	125	
6	128	
6	125	
7	126	
7	129	
8	129	
8	132	
9	131	
10	131	24-hour urine collection for salt wasting
11	134	2 L of 1.8% saline, Ure-Na 15 g bid, fludrocortisone 0.1 mg
12	131	
13	133	
14	138	
14	137	
15	142	Started salt tabs 3 g tid, increased fludrocortisone to 0.3 mg, stopped 1.8% saline and Ure-Na 15 g bid
15	136	
16	141	
16	135	
17	133	
17	136	
18	140	Salt tabs increased to 5 g tid, fludrocortisone at 0.3 mg
18	137	
19	138	
22	140	
29	140	

## Discussion

The differential diagnosis in our case included CSW, SIADH, diabetes insipidus (DI), adrenal insufficiency, severe hypothyroidism, and osmotic diuresis from medication usage.

DI should be considered in a patient with abnormal serum sodium concentration in the setting of intracranial pathology. However, this was effectively ruled out in our patient given the low plasma sodium concentration because DI is characterized by inappropriately elevated serum sodium concentrations (i.e., hypernatremia) as opposed to hyponatremia observed in this case. Furthermore, DI, whether central or nephrogenic, is characterized by reduced urinary sodium concentration, which was not the case in our patient.

Determining the underlying cause of hyponatremia is crucial in medical management because treatment options often depend on the etiology. Several key possibilities should be considered in patients presenting with acute or chronic hyponatremia. Hormone imbalances including adrenal insufficiency and hypothyroidism have been shown to cause hyponatremia and should be evaluated as part of the workup for any patient with new hyponatremia. In addition, SIADH and CSW should be considered in patients with hyponatremia associated with intracranial pathology, as was the case in our patient.

Both primary and secondary adrenal insufficiencies are known to cause hyponatremia associated with renal salt wasting (i.e., elevated urinary sodium concentrations, particularly above 40 mmol/L). Secondary adrenal insufficiency resulting from inadequate ACTH production is characterized by normovolemic hyponatremia given the intact production of mineralocorticoid. However, primary adrenal insufficiency is characterized by a failure to produce both mineralocorticoids and glucocorticoids resulting in hypovolemia often accompanied by hypotension [2]. Both of these disease states can be notoriously difficult to differentiate from other possible causes of hyponatremia without further workup because they are often clinically indistinguishable from other causes of hyponatremia, including SIADH and CSW, and thus should be ruled out before a comprehensive treatment plan can be initiated. While suspicion for primary adrenal insufficiency was higher for this patient given the clinical evidence for hypovolemia and relative hypotension that persisted throughout the patient’s hospital course, a normal ACTH stimulation test indicated that adrenal insufficiency was not the primary cause of the hyponatremia and urinary sodium losses seen in this patient.

Severe hypothyroidism has also been shown to cause hyponatremia. Although the mechanism for this has not been elucidated, it is likely that a decrease in cardiac output results in both a decrease in glomerular filtration rate and an increase in antidiuretic hormone (ADH) release via carotid baroreceptor reflex [3]. However, hypothyroidism as a contributing factor to this patient’s hyponatremia was easily ruled out with a normal TSH level.

SIADH is the most common cause of hyponatremia in the inpatient setting and leads to impaired water excretion as a result of inappropriately elevated levels of ADH and/or the inability to suppress the production of ADH. Common etiologies of SIADH include pulmonary disease, a broad range of intracranial pathology, malignancy, and drug-induced [4]. SIADH should be considered in patients with elevated urine osmolality despite the setting of hyponatremia and is typically characterized by high urinary sodium concentration and clinical signs of normovolemia or even expansion of the extracellular compartment [5].

CSW is a potential cause of hyponatremia in patients with neurologic disease, most commonly those with subarachnoid hemorrhage. CSW was first described in 1950 by Peters et al. [1] who reported three patients with intracranial disorders and associated hypovolemic hyponatremia and clinical symptoms of diuresis, natriuresis, and dehydration. Both CSW and SIADH have similar features and laboratory signs but different treatment regimens, making diagnosis and successful management critical. CSW is diagnosed in patients who have evidence of hypovolemia, hyponatremia with low plasma osmolality, and inappropriately elevated urine osmolality, as well as an elevated urine sodium concentration with evidence of net negative sodium balance and low serum uric acid concentration due to urate wasting in urine [6].

The etiology of CSW is not well understood but may develop as a protective mechanism in response to excessive increase in intracranial pressure, vasospasm in subarachnoid hemorrhage, or meningitis [7]. One hypothesis suggests that natriuretic factors such as BNP are involved, causing impairment in renal tubular sodium reabsorption and inhibition of renin release leading to an increase in sodium excretion and urine volume [8]. Another hypothesis suggests that CSW is caused by sympathetic neural input impairment to the juxtaglomerular apparatus that reduces proximal tubule sodium, urate, and water reabsorption and decreases renin and aldosterone release [9].

Only a handful of CSW cases in adult patients with central nervous system (CNS) disease due to TB have been reported in the literature, mostly endemic to Southeast Asia where TB is more common. CNS disease occurs in approximately 1-5% of TB cases, but the clinical presentation may be non-specific leading to potentially delayed or missed diagnosis. Patients typically present with constitutional and neurologic symptoms with a history of weight loss, fever, extremity or facial weakness, tremor, headache, altered consciousness, or personality changes [10]. Complications of CNS TB include hyponatremia, hydrocephalus, stroke, cranial nerve palsies, epileptic seizures, DI, myeloradiculopathy, and hypothalamic syndrome due to tuberculous meningitis, tuberculoma, or spinal arachnoiditis [11]. Because severe hyponatremia causing AMS may mask the clinical symptoms and signs of CNS TB, a high index of suspicion for the possibility of TB must be applied. In this patient, the CT scan and MRI of the head showed a 7 mm enhancing lesion in the left thalamus/midbrain junction with surrounding vasogenic edema and mass effect. As in this patient, a definitive diagnosis of tuberculoma is established via needle biopsy of the CNS lesion for histopathology and acid-fast bacilli stain and culture, and further workup including chest imaging for active pulmonary manifestations should be completed [12].

Previously reported cases of CSW in the setting of TB meningitis initially considered SIADH as the main cause for hyponatremia, but patients did not correct with fluid restriction and 3% hypertonic saline, leading to further loss of sodium in the urine [13,14]. The fractional excretion of uric acid (FEUA) is also used as a marker to distinguish CSW as increased levels of FEUA persist after correction of hyponatremia by water restriction and salt supplementation [15]. Recent literature has suggested that CSW is the predominant cause of hyponatremia in patients with TB meningitis over SIADH or any other causes and should be treated appropriately as soon as possible when diagnosed [16]. Our patient was also initially treated with 3% hypertonic saline and a 1.5 L fluid restriction to help correct hyponatremia with the possibility that it may be due to SIADH or CSW. However, the patient lost 15 g of sodium chloride excreted in urine over 24 hours further confirming the likely etiology of hyponatremia was due to CSW. Salt repletion with oral salt tablets was initiated to help correct the electrolyte imbalances.

Treatment also includes fludrocortisone as CSW causes inhibition of the renin-angiotensin-aldosterone system. A recent study by Misra et al. showed that patients with CSW treated with oral fludrocortisone normalized serum sodium levels significantly earlier than those receiving normal saline alone; however, this did not affect mortality or development of disability [17]. This case report confirms that the use of fludrocortisone titrated up to 0.3 mg helped stabilize the patient’s severe hyponatremia and treat the possible underlying adrenal insufficiency, as evidenced by the patient’s baseline hypotension with blood pressures ranging from low 80-100/50-70 mmHg throughout the hospital course.

The use of urea, an osmotic diuretic that increases the excretion of free water by the kidneys, has been supported for the treatment of SIADH. The addition of urea in a patient whose primary pathology is inappropriate natriuresis only worsens volume status without improving sodium balance. However, in the case of SIADH, osmotic diuresis with urea helps prevent the expansion of the extracellular space while allowing for more liberal fluid restriction goals without the risk of volume contraction associated with oral salt tablets. Urea treatment in patients with SIADH has been shown to be safe and well-tolerated. However, the same retrospective study that found evidence in support of urea treatment for SIADH identified a contraindication for urea in cases of hypovolemic hyponatremia [18]. The clinical deterioration of this patient following administration of urea suggests that osmotic diuresis in the setting of continued salt wasting resulted in a symptomatic decline in plasma sodium levels and extracellular volume that only improved with administration of 1.8% saline solution along with oral salt tablets. This finding in conjunction with elevated 24-hour urine sodium excretion offers strong evidence of a continued net negative sodium balance and significant renal salt wasting in support of CSW over SIADH.

## Conclusions

This case provides more insight into a unique report of CSW caused by CNS TB and highlights the importance of recognizing CSW early in a patient presenting with severe hyponatremia. This helps initiate the correct treatment regimen. Although CSW is difficult to distinguish from SIADH, 24-hour urine sodium excretion levels and FEUA should be used to help determine the etiology of hyponatremia. Treatment should include fludrocortisone, oral salt repletion, 3% saline, and fluid restriction to help correct severe hyponatremia and prevent further neurologic complications in patients with CNS TB.
